# 

**Published:** 1886-03

**Authors:** 


### CONTENTS.

COMMUNICATIONS.

Heredity.......... 109
A Bill to Insure the Better Education of Practitioners of Dental Surgery etc.,.......... 122

PROCEEDINGS.

Illinois State Dental Society.......... 126
Chicago Dental Society.......... 152
American Dental Association.......... 154

SELECTIONS.

The Etiology of Rachitis.......... 128
Indigestion and Its Treatment.......... 133
Indigestion in Children.......... 136
Observations on the Oleates.......... 138
Preserving Cocaine Solutions.......... 139
A Reliable Remedy for Ozaena.......... 140
A Remarkable Instance of Longevity.......... 141
Therapeutical Hints.......... 142
Napelline in the Treatment of Odontalgia.......... 143
Salicylate of Cocaine in the Treatment of Trigeminal Neuralgia..... 143
Deutche Monatschrift fur Zahnheilkunde.......... 144
Jealousy Among Doctors.......... 149
Is Cancer Hereditary?.......... 149
Pilocarpin in Toothache.......... 150
A Question of Sanity.......... 150
Chalfant's Pardon.......... 151

SALMAGUNDI.......... 155

EDITORIAL.

Bibliographical.......... 159
Alabama State Dental Society.......... 160

